# Geologic evolution of the Moon studied through young lunar basaltic volcanism

**DOI:** 10.1038/s41467-026-72083-7

**Published:** 2026-05-11

**Authors:** Jingyou Chen, Fanglu Luo, Huicun He, Lauren Galien, Bre H. Oliveira

**Affiliations:** 1 State Key Laboratory of Deep Earth Processes and Resources, Guangzhou Institute of Geochemistry, Chinese Academy of Sciences, Guangzhou, China; 2 Center for Advanced Planetary Sciences (CAPS), Guangzhou Institute of Geochemistry, Chinese Academy of Sciences, Guangzhou, China; 3 Planetary Environmental and Astrobiological Research Laboratory, School of Atmospheric Sciences, Sun Yat-sen University, Zhuhai, China; 4 Key Laboratory of Planetary Science and Frontier Technology, Institute of Geology and Geophysics, Chinese Academy of Sciences, Beijing, China; 5 Department of Civil & Environmental Engineering & Earth Sciences, University of Notre Dame, Notre Dame, IN USA; 6 Department of Earth and Environmental Sciences, University of Manchester, Manchester, UK

**Keywords:** Planetary science, Early solar system

## Abstract

Despite the Moon being Earth’s nearest celestial neighbor, many questions about its geology remain unanswered. Recently, China’s Chang’e-5 (2020) and Chang’e-6 (2024) missions returned new basaltic samples, allowing us to make major advances in understanding the Moon’s geologic evolution.

## Background

Since the dawn of humanity, our Moon has captured human interest, particularly with regard to its origin and evolution. Studying the Moon’s geological history also informs our understanding of the origin and evolution of Earth and the early dynamics of the inner Solar System. Today, the most widely accepted theory for the Moon’s formation is the giant-impact hypothesis: A Mars-sized impactor (commonly called Theia) collided with the proto-Earth and the resultant debris coalesced to form the Moon-Earth system^1^. The enormous thermal energy induced by this event caused the Moon to be either fully or partially molten, a state referred to as the ‘lunar magma ocean’(LMO). Gradually, the LMO cooled and differentiated based on density disparities between the crystallizing mineral phases and the remaining melts. First, Mg-rich (i.e., olivine and orthopyroxene) and Fe-rich (i.e., clinopyroxene) minerals crystallized and sank to form the mantle. Then, after 75–80% of the LMO had solidified, plagioclase crystallized and floated to the surface, forming the lunar anorthositic crust. Between these two layers, the last residual melt became enriched in incompatible elements, termed urKREEP (where KREEP is an abbreviation of K, rare earth elements, and P).

**Fig. 1:**
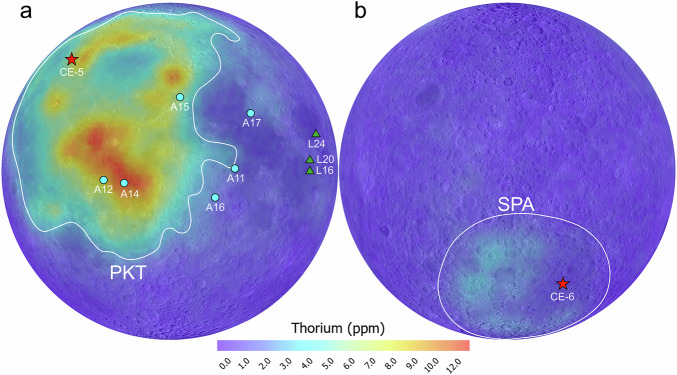
Global thorium (Th) distribution maps of the Moon. **a** Moon’s nearside. **b** Moon’s farside. Sampling sites from the CE (red stars), Apollo (blue circles), and Luna (green triangles) missions are marked. The white outlines denote the Procellarum KREEP Terrane (PKT) on the nearside and the South Pole–Aitken (SPA) Basin on the farside. Data source from ref. ^23^.

Following LMO differentiation, various processes—including heating, decompression, and the addition of volatiles (e.g., H_2_O)—caused partial melting of the mantle to melt and extrusion onto the lunar surface, creating mare basalts. These mare basalts provide direct information about the lunar interior and volcanic history. Decades of analysis of basalt samples returned by the Apollo missions have established a strong baseline for understanding the lifespan and dynamic mechanisms of lunar volcanism, as well as the composition and structure of the Moon’s interior. However, as Apollo samples were collected from only seven limited regions on the Moon’s equatorial nearside (Fig. 1a), these classical theories may not be fully informed. More recent evidence from the CE-5 and CE-6 basalt samples, obtained from beyond the Apollo landing sites, has prompted reconsideration of lunar volcanism and the information it carries about the Moon’s evolution.

## Lunar evolution as seen through the Apollo samples

Early telescopic observations of the Moon only provided a blurry outline of its geology, revealing regions of low and high albedo, later respectively termed as the maria and highlands. In 1969, the Apollo 11 mission returned the first lunar samples to Earth, confirming that the dark maria are predominantly composed of basaltic rocks. Since then, studies of these basaltic samples have yielded insights into the duration, timing, thermal mechanisms, and mantle sources of lunar volcanism.

Mare basalts are commonly divided into three subgroups based on their Ti abundance^2^. During magmatic processes, Ti behaves as an incompatible element, becoming enriched in the liquid melt (late cumulates), rather than entering early-crystallizing mineral phases. Thus, variations in Ti concentrations among mare basalts reflect differences in the mantle source regions. For example, low-Ti basalts are thought to originate from a Ti-poor mantle source (i.e., a peridotitic mantle), whereas very low-Ti basalts are inferred to derive from an even more Ti-depleted mantle source at greater depths^2^. This supports the concept of a layered lunar interior, consistent with the predicted crystallization sequence of the LMO^3^, in which the early-forming, denser crystals that sank contained less Ti, while the later-formed and shallower cumulates contained higher Ti abundances. In this framework, high-Ti basalts cannot be solely generated by melting a Ti-poor peridotitic mantle. Instead, they require the incorporation of Ti-rich materials, referred to as the ilmenite-bearing cumulates (IBCs)^3^. These IBCs formed at shallow depths after ~90% solidification of the LMO^3^. Because these IBCs are greater than the underlying layers, they are thought to have sunk via a process known as mantle overturn, potentially reaching the mantle–core boundary^4^. The inclusion of mantle overturn processes further refines the LMO model and provides insights into the evolution of the Moon’s interior.

The absolute ages and chemical compositions of Apollo basalts place important constraints on the duration and thermal dynamics of lunar volcanism. Apollo basalts formed approximately 3.1–3.9 Ga ago^5^. This is consistent with statistical crater-counting model ages of global lunar volcanic units derived from lunar chronology functions calibrated using Apollo sample ages^6^. The clustering of Apollo basalt ages, together with remote-sensing observations of mare regions, led to the suggestion that lunar volcanism largely ceased after ~3.0 Ga, despite the discovery of rare irregular mare patches with a younger age of ~100 Ma^7^.

Lunar volcanism may be triggered by a wet mantle reservoir, as the addition of H_2_O lowers the melting point. However, the H_2_O abundance in the lunar interior appears to be heterogeneous. Some Apollo basalt samples indicate a relatively high H_2_O reservoir (~100 ppm), suggesting a wet mantle^8^, while others sample a low H_2_O reservoir (~1 ppm) on behalf of a dry mantle^9^. Consequently, high H_2_O reservoirs appear to play a critical role in triggering lunar volcanisms. Notably, many Apollo basalts exhibit a geochemical KREEP component, as seen in the isotopic signatures of their mantle sources (i.e., high ^238^U/^204^Pb and ^87^Rb/^86^Sr ratios and low ^147^Sm/^144^Nd ratios). Global Th abundance obtained by the Lunar Prospector gamma ray spectrometer reveals that all Apollo landing sites are within or near the Procellarum KREEP Terrane (PKT), a region known for its high Th abundance (Fig. 1a). As KREEP is rich in heat-producing elements like Th and U, it can act as a heat source to lower the melting temperature of rocks. As a result, KREEP has been proposed as a key factor in prolonging lunar volcanic activity. Nevertheless, whether such mechanisms also drove younger episodes of lunar volcanism remains uncertain.

## Recent insights from returned young CE basalts

In 2021, China’s CE-5 mission successfully returned ~1.731 kg lunar soil from a volcanic area within the PKT on the Moon’s nearside (Fig. 1a). The CE-5 basalts are not only collected from a previously unsampled region, but are also chemically distinct from the Apollo samples. Dated to ~2.0 Ga, they represent the youngest volcanic rocks ever collected from the Moon^10^, thereby making them crucial for investigating the thermal drivers of younger lunar volcanism. Surprisingly, isotopic compositions (e.g., Sm-Nd, U-Pb, and Rb-Sr) suggest their mantle sources contain little to no KREEP components^11,12^, compared to the majority of Apollo basalts. In 2024, CE-6 returned ~1.935 kg of lunar soil from the South Pole-Aitken (SPA) basin on the lunar farside (Fig. 1b). Like the CE-5 basalts, the CE-6 basalts are young (~2.8 Ga) and KREEP-poor^13–15^. Collectively, these KREEP-poor mantle sources indicate that KREEP is not an essential precondition for a prolonged period of lunar volcanism. Additionally, the mantle sources of the CE-5 and CE-6 basalts are estimated to be dry (only ~1–5 ppm H_2_O abundance)^16,17^, which contradicts the viewpoint that a wet lunar mantle caused younger episodes of lunar volcanism.

Both the CE-5 and CE-6 basalts belong to the low-Ti group, and they could derive from the IBCs layer at depths of ~60–100 km^8,18,19^. Their source depths are significantly shallower than those of Apollo low-Ti basalts (~500 km), indicating that they may be a new type of lunar basalt^19^. Also, these IBCs would have been shallower than their Apollo counterparts, implying that not all IBCs sank into the deeper mantle during the overturn process.

The CE-6 basalts were the first lunar samples collected from the farside, providing the first information about the farside mantle. Geochemical features (e.g., KREEP abundance and H_2_O) of the farside mantle revealed by CE-6 basalts are distinct from those of nearside samples^10,13^. This either supports a compositionally heterogeneous lunar interior after the cooling of the LMO or suggests that the Moon’s nearside and farside mantle may be compositionally distinct, possibly due to an asymmetric crystallization of the LMO)^13^. Given that the CE-6 landing site lies within the largest SPA basin, the observed geochemistry might also represent modifications to the mantle source caused by the SPA impact event^13,15^. Additionally, CE samples provide indirect information on the Moon’s external impact environment. Age data from the CE-5 and CE-6 basalts fill a ~1.0–3.0 Ga gap in the lunar crater chronology, suggesting that the frequency of impact events became relatively steady at ~2.83 Ga^20^. CE-6 samples also enabled age dating of the Apollo and SPA basins^21,22^, which improves our understanding of the frequency of small celestial bodies hitting the early Moon.

## Objectives for future lunar explorations

CE lunar samples, in terms of their young ages and unique mantle compositions, have provided new constraints on the Moon’s geological past; most notably on the thermal drivers of lunar volcanism, and the composition and structure of the Moon’s interior. A significant milestone in lunar exploration, CE-6 delivered the first sample-based information about the farside volcanism, mantle composition, and impact history. However, the limited number of farside samples currently available is insufficient to fully characterize lunar geology, particularly given the pronounced differences in volcanism, crustal thickness, and the distribution of heat-producing elements between the nearside and farside^6^. To achieve a more global understanding of the Moon, future missions must prioritize additional farside sample returns. NASA’s upcoming Artemis III mission plans to collect samples from the SPA region to further investigate the Moon’s farside geology. In parallel, the China National Space Administration’s CE-7 and CE-8 missions aim to land within lunar polar regions to search for water, a critical resource for supporting sustained human presence on the Moon.
